# Prevalence and molecular insights of *Mycobacterium avium* subsp. *paratuberculosis* in Sudanese Cattle: implications for control and public health

**DOI:** 10.1007/s42770-025-01781-z

**Published:** 2025-10-07

**Authors:** Sanaa M. Idris, Wisal A. Elmagzoub, Mohamed E. Mukhtar, Julius B. Okuni, Lonzy Ojok, Enass M. Abdalla, Sulieman M. El Sanousi, Ahmad Amanzada, Uwe Truyen, Ahmed Abd El Wahed, ElSagad Eltayeb, Ahmed A. Gameel, Kamal H. Eltom

**Affiliations:** 1 Institute of Animal Hygiene and Veterinary Public Health, Faculty of Veterinary Medicine, Leipzig University, An den Tierkliniken 1, Leipzig, 04103 Germany; 2 Department of Animal Health and Safety of Animal Products, Institute for Studies and Promotion of Animal Exports, University of Khartoum, Shambat 13314, Khartoum North, Sudan; 3 Department of Pathology, Faculty of Veterinary Medicine, University of Khartoum, Shambat 13314, Khartoum North, Sudan; 4 Department of Biology and Biotechnology, College of Applied and Industrial Sciences, University of Bahri, Khartoum North, Sudan; 5 Department of Agricultural Extension and Rural Development, Faculty of Agriculture, University of Khartoum, Shambat 13314, Khartoum North, Sudan; 6 College of Veterinary Medicine, Animal Resources and Biosecurity (COVAB), Makerere University, P. O. Box 7062, Kampala, Uganda; 7 Department of Pathology, Faculty of Medicine, Gulu University, P.O.Box 166, Gulu, Uganda; 8 Department of Microbiology, Faculty of Veterinary Medicine, University of Khartoum, Shambat 13314, Khartoum North, Sudan; 9 Department of Gastroenterology and Gastrointestinal Oncology, University Medical Centre Göttingen, Göttingen, Germany; 10 Faculty of Medicine, Al Neelain University/Ibn Sina Specialised Hospital, Alamarat 12217, Street 17-21, Khartoum, Sudan

**Keywords:** *Mycobacterium avium* subsp. *paratuberculosis*, Recombinase aided amplification assay, Cattle

## Abstract

Paratuberculosis (PTB) is a chronic intestinal disease affecting ruminants and somenon-ruminants, caused by *Mycobacterium avium* subsp. *paratuberculosis* (MAP). In the Sudan, published data on the incidence and prevalence of PTB are Limited. we detected MAP in human patients with gastrointestinal complaints highlights its zoonotic potential and raises public health concerns. This study aimed at assessing PTB prevalence in cattle and identifying risk factors for MAP infection as well as investigating the phylogeny of MAP circulating in the Sudan. Both serum and faecal samples were collected from the same individual animals of 810 cattle in 153 herds in five states spanning three regions (Southern, Northern, and Central) of the country. ELISA was used to detect MAP antibodies in sera, while faecal samples were tested for MAP DNA using a recombinase aided amplification (RAA) assay and cultured for MAP isolation followed by partial sequencing of MAP insertion sequence 1311 with subsequent phylogeny analysis. At the animal level, the apparent prevalence was 5.0% for ELISA and 4.2% for RAA, with true prevalence estimates of 8.5% and 4.8%, respectively. At the herd level, apparent prevalence was 28.2% for ELISA and 22.3% for RAA, while true prevalence reached 54.2% for ELISA and 24.9% for RAA. Significant (*P* < 0.05) risk factors for MAP infection included exposure to wild animals and high rainfall. Phylogenetic analysis of the Sudanese MAP isolates revealed close relatedness to type S (I/III) strains worldwide suggesting a shared evolutionary origin. The present study provides baseline data on PTB prevalence and risk factors in Sudanese cattle, emphasising the role of environmental and management factors in disease dynamics. These findings highlight the necessity of adopting targeted control strategies to reduce MAP impact on cattle and other animals as well as to prevent its potential public health hazard.

## Introduction

Paratuberculosis (PTB) or Johne’s disease is a chronic infectious disease caused by *Mycobacterium avium* subsp. *paratuberculosis* (MAP). It primarily affects ruminants, such as cattle, sheep, and goats, but has also been reported in some non-ruminant species [1, 2]. PTB is characterized by progressive granulomatous inflammation, leading to chronic diarrhoea, weight loss, decreased milk production, and eventually death in severe cases [3]. The disease not only impacts the health and productivity of affected animals, but also causes significant economic losses for the livestock industry worldwide, particularly in terms of reduced yields, increased veterinary costs, and premature culling of infected animals [4–7].

The aetiological agent of PTB, has been detected in and isolated from patients suffering from Crohn’s disease and other human diseases [8–11]. Thus, MAP can be considered a public health hazard (Bharathy et al., 2017).

Managing the disease is challenging due to its prolonged incubation period, during which infected animals, while asymptomatic, shed the pathogen and act as silent carriers. This makes it difficult to identify and isolate MAP to prevent transmission, a problem that is further exacerbated by the lack of rapid and accurate diagnostic tests [12]. Moreover, environmental persistence of MAP further complicates its control, as the bacterium can survive for extended periods in the soil and water under favourable conditions (Derakhshandeh et al., 2018). The risk factors that have been reported to influence MAP transmission include poor biosecurity practices, overcrowding, exposure to contaminated water sources, and environmental conditions, such as high rainfall, which promotes pathogen dissemination [13–17].

Globally, numerous studies have been conducted to estimate the prevalence and epidemiology of PTB and associated risk factors, as well as to characterize the genetic diversity of MAP strains [14, 18–28]. However, in the Sudan, a country with a significant cattle population and a heavy reliance on livestock for food security and economic stability [29], data on PTB prevalence and risk factors remain scarce [30, 31]. The limited availability of epidemiological information hampers efforts to understand the impact of the disease on the national livestock industry and to develop targeted interventions.

This study investigated the prevalence and risk factors of PTB in cattle across Southern, Northern, and Central regions of the Sudan, using ELISA and molecular diagnostic methods to detect MAP infection at individual and herd levels. It also explored the phylogenetic relationships of Sudanese MAP isolates to global strains to provide valuable baseline data on PTB epidemiology in the Sudan.

## Materials and methods

### Study area

**Fig. 1:**
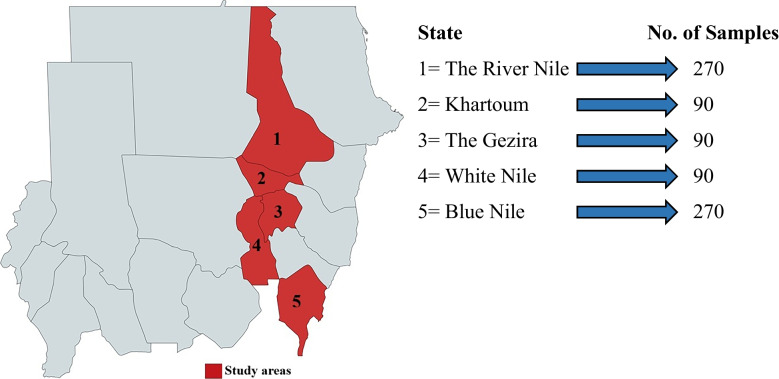
Map of the Sudan showing the States of samples collection (in red colour). This map is for demonstration purpose only and does not represent the official map of the Sudan. It was created from https://mapchart.net/world.htm

This study was conducted across three regions of the Sudan to assess the prevalence of PTB and its associated risk factors in cattle. The study covered the southern region, represented by Blue Nile State; the northern region, represented by the River Nile State; and the central region, encompassing Khartoum, the Gezira, and White Nile States (Fig. 1). The samples were collected over two years. Grazing systems differed across regions, ranging from semi-intensive in the central region to semi-extensive in parts of the southern and northern regions. Environmental conditions also vary, with the southern region experiencing high rainfall, while the northern and central regions face prolonged dry periods (El Moghraby, 2003).

### Collection of samples

The animal-level sample size was calculated using the Cochran’s formula [32]

$$\text{Sample Size}=\text{Z}^2\times\text{p}\times(1-\text{p})/\text{C}^2$$

A Z-value of 1.645 was applied, corresponding to a 90% confidence level. In the absence of reliable prior prevalence data, an expected prevalence (p) of 50% was adopted, as recommended by Cochran. This conservative estimate yields the largest possible sample size, thereby enhancing statistical power. A margin of error (C) of 5% was chosen to ensure accurate prevalence estimation.

Based on this calculation, the minimum required sample size was 270 animals per region. Sampling was conducted across three regions: Blue Nile State, River Nile State, and a combined cluster of three central states (Khartoum, Gezira, and White Nile), resulting in a total of 810 animals sampled.

Accordingly, 810 faecal and blood samples across 153 herds were collected using stratified random sampling. Stratification was based on geographic region, accessibility, and herd size. Within each selected herd, animals were randomly sampled in proportion. Samples were collected after obtaining consent of animal owners and permission from the federal and local veterinary authorities, and the ethical approval was obtained from the Faculty of Veterinary Medicine, University of Khartoum (FVM 1–1-2021). From each animal, after taking all necessary precautions, bout 4 ml of blood was withdrawn by jugular venepuncture into plain tubes, while a faecal sample was collected directly from the rectum and put into screw- capped plastic vials and transferred in mobile refrigerator to the laboratory of the Institute for Studies and Promotion of Animal Exports, University of Khartoum. The serum was separated by centrifugation and all samples were stored at – 20 °C until needed.

### Detection of MAP antibodies

A commercial kit (IDEXX Paratuberculosis screening, IDEXX Laboratories, Inc., Westbrook, USA) of an indirect ELISA assay was used to detect antibodies against MAP. A pre-incubation step of the serum samples and controls in a dilution buffer (1:20) containing *Mycobacterium phlei* was included in the assay to remove cross-reacting antibodies of other mycobacteria. Briefly, according to the manufacturer instructions, 100 µl of the diluted sample were dispensed into a 96-well microtiter plate of ELISA kit and incubated at RT for 45 min, then the coated plate was washed by diluted washing buffer (1:20). The diluted conjugate (1:100) was added to each well and incubated at RT for 30 min. The second washing was performed before adding 100 µl of Tetramethylbenzidine (TMB) substrate before incubation for 10 min. In the last step 100 µl of the stop solution was added. The optical density (OD) of samples and controls was measured at 450 nm, and the results were expressed as ratio of samples to positive (S/P), which were calculated using samples OD, mean OD of positive control and the OD of the negative control. The samples were interpreted as positive if the S/P ratio was 55% or higher.

### Detection of MAP DNA

#### Extraction MAP DNA

The Mobile Suitcase Laboratory developed by Abd El Wahed et al. [33] was used for extraction and detection of MAP DNA in faecal samples. MAP DNA was extracted according to method described by Hansen et al. [34] using the SpeedXtract kit (Qiagen, Hilden, Germany). Briefly, 100 mg of faecal samples were added to 500 μl of lysis buffer in a Precelly’s SK 38 (Bertin Corp., Rockville, MD, USA), then vortexed for 1 min. Then, 60 μl well mixed magnetic beads were added to the sample and vortexed for 10 s. A heat block was used to incubate samples at 95 °C for 15 min with vortexing every 2 min. The sample tubes were placed on a magnetic rack for 2 min, then 10 μl of the clear solution were transferred to a 0.5 ml tube containing 40 μl of molecular biology grade water (1:5).

#### Recombinase aided amplification (RAA) assay

The isothermal technique was used to detect MAP DNA in faecal samples based on a method described by Hansen et al. [35]. Briefly, 13 μl of oligo mix (primers and probe) were added to 29.5 μl of rehydration buffer and 5 μl of the DNA sample into a freeze-dried reaction pellet of RAA kit (QT Biotech Co., Ltd. Wuxi China) and 2.5 μl of magnesium acetate was dispensed into the lid of each tube. For each run positive (molecular standard) and negative controls were used for results interpretation. Centrifugation and vortexing were done for sample tubes before being placed into the tube scanner (Axxin, Fairfield, Australia) and after 3 min of the beginning of the reaction. The results were seen in real time on the machine screen.

#### Risk factors associated with paratuberculosis in cattle

A structured check list was used to record some potential risk factors (Appendix 1) of PTB infection. The check list was designed to collect information about the environment, type of soil, grazing system, exposure to wild animals and to other ruminants.

#### Isolation MAP

The faecal samples for MAP isolation were selected from animals that tested positive for MAP DNA in RAA or MAP antibodies in ELISA; these were also positive for acid fast bacilli in faecal smears stained with Ziehl-Nielsen stain.

The faecal samples were decontaminated using sterile solution of 0.75% hexadecyl pyridinium chloride (HPC) following standard decontamination protocols before being inoculated on Middlebrook 7H11 (Merck, Darmstadt, Germany) agar slants supplemented with vancomycin, nalidixic and amphotericin B (VAN), oleic acid- albumin-dextrose-catalase (OADC) and mycobactin J (2 mg/L, Allied Monitor, USA). The inoculated slants were incubated at 37 °C, checked for visible growth after 4 weeks and then every month for up to 20 months. Positive growth of MAP was confirmed by RAA, PCR and partial sequencing of MAP IS 1311.

### Sequencing and phylogenetic analysis of *mycobacterium avium* subsp. *paratuberculosis* (MAP) isolates from cattle

#### Extraction of MAP DNA from culture

The wire loop was used to scrape slant surfaces of MAP positive cultures. The colonies were emulsified in Tris EDTA buffer (pH 8.0) in 2- ml Eppendorf tubes and heated at 95 °C for 15 min, as described before (Sweeney et al., 2016).

#### Nested- polymerase chain reaction (nPCR) of the IS1311 element

For partial amplification of the MAP IS 1311 by PCR, the assay described by Whittington et al. (Whittington et al., 1998) was followed. The Maxime premix (Intron Seoul, Korea) was adjusted to 20 µl total reaction volume with 13 µl molecular biology grade H_2_O, 1 µl (10 pMol/µl) of each primer and 5 µl of DNA template for the first PCR round. For the second PCR round, 17 µl of H_2_O and 1 µl of each primer as well as the PCR product were used. The primer set M56 F: 5`- GCG TGA GGC TCT GTG GTG AA-3`; M62: 5`- GCC TAT TTG CAC GGC ACC TC-3` was used for the first PCR, and the set M57 5`- GAT TGG TCG GCT GAA TCG GA-3`; M63 5`- GAT CCC TTG GGC ACC TGG GC-3`) was for the second PCR round. The following conditions were used for both PCR rounds: one cycle of denaturation at 94 °C for 2 min followed by 37 cycles each composed of denaturation at 94 °C for 30 s, annealing at 62 °C for 15 s and extension at 72 °C for 1 min. The PCR amplification products were evaluated by electrophoresis at 100 V in 1% agarose gels stained with SYBR safe gel stain (Invitrogen, Carlsbad, CA, USA), using100 bp DNA ladder as size marker.

#### Sequencing of IS1311 gene and phylogenetic analysis

The amplification products of the nested PCR of the MAP IS1311 were sent to a commercial company (Macrogen-Europe, Amsterdam, the Netherlands) for sequencing using the amplification primers. Sequences with high quality were edited using BioEdit programme (Hall, 1999) and the edited sequences were aligned to corresponding sequences of the available MAP strains in NCBI GenBank using the Basic Local Alignment Search Tool (BLAST).

Phylogenetic trees were constructed using the sequences of the Sudanese MAP isolates with those of strains worldwide using the Geneious programme version 10.0 (Biomatters, http://www.geneious.com), setting the bootstrap replicates to 500.

### Data analysis

EPITOOLS programme (http://epitools.ausvet.com.au) was used to estimate the true prevalence of the ELISA and RAA results with a 90% confidence level. True prevalence refers to the actual proportion of infected individuals in the population, accounting for potential misclassification due to the sensitivity and specificity of the diagnostic tests. The sensitivity and specificity of the ELISA KIT (McCormick et al., 2010) and RAA [35] were considered in the calculation to adjust for false positives and false negatives, providing a more accurate estimate of disease occurrence.

Nominal logistic regression was used to identify associations between recorded possible risk factors and herd MAP status. A herd was classified positive if one animal was positive by ELISA and/or RAA. Variables recording *P* < 0.05 were considered significant.

## Results

**Fig. 2:**
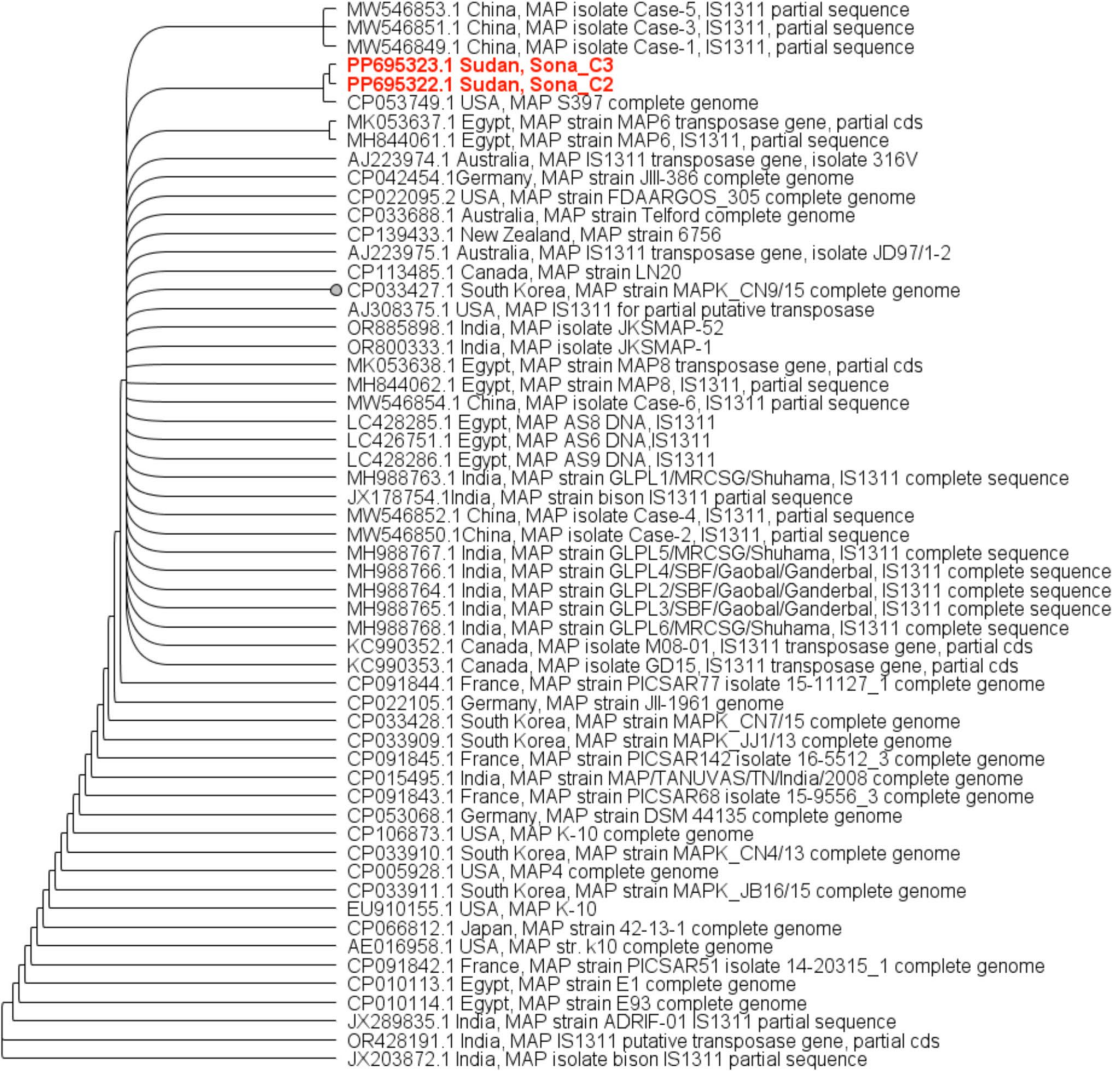
Maximum Likelihood phylogenetic tree based on partial sequence of 1311 insertion element of the Sudanese MAP cattle (Sona-C2 and Sona- C3) strain with corresponding sequence of other strains worldwide. Sona-C2 and Sona C3 clustered with strains such as Telford from Australia, MAP S397 from the USA, MAP 386 from Germany, and MAP 6756 from New Zealand, which were classified as type S strains. The tree was created by Geneious version 10.0 (Biomatters, http://www.geneious.com)

A total of 810 cattle, comprising both crossbred (local x Friesian) and local Zebu breeds from 153 herds across five states in three selected regions, were tested for MAP antibodies using ELISA and for MAP DNA using RAA. Antibodies against MAP were detected in 41 (5.1%) of the 810 serum samples. The average apparent and true seroprevalence of PTB at the animal level was 5.0% and 8.5%, respectively, while at the herd level, it was 28.2% and 54.2%, respectively (Table 1). Seroprevalence was highest in the Gezira (13.9%) and Blue Nile (11.7%) States, and lowest (4.4%) in the River Nile State (Fig. 2).

**Table 1:** Prevalence of anti-MAP antibodies of cattle in five states of the Sudan

State	No. of tested animals(n)	No. of positive animal (n)	Apparent prevalence at animal level (%)	True prevalence at animal level (%)	No. of tested herd (n)	No. of Positive herds (n)	Apparent prevalence at Herd level (%)	True prevalence at herd level (%)
Khartoum	90	3	3.3	5.1	46	3	6.5	11.4
White Nile	90	5	5.5	9.5	8	4	50.0	97.2
The Gezira	90	7	7.7	13.9	13	6	46.1	89.6
Blue Nile	270	18	6.6	11.7	57	13	23.0	43.6
River Nile	270	8	2.9	4.4	29	8	27.5	53.0
	810	41	Av. 5	Av. 8.5	153	34	Av. 28.2	Av. 54.2

Out of 810 faecal samples, 35 (4.3%) tested positive for MAP DNA by RAA. The average apparent and true prevalence of PTB at the animal level was 4.2% and 4.8%, respectively, while at the herd level, it was 22.3% and 24.9%, respectively (Table 2). The Gezira and Blue Nile States also exhibited higher prevalence rates compared to other States (Fig. 2).

**Table 2:** Prevalence of MAP DNA detected by the recombinase aided amplification assay (RAA) in faecal samples of cattle in five states of the Sudan

State	No. of tested animals (n)	No. of positive animals (n)	Apparent prevalence at animal level (%)	True prevalence at animal level (%)	No. of tested herds (n)	No. of Positive herds (n)	Apparent Prevalence at herd level (%)	True Prevalence at herd level (%)
Khartoum	90	1	1.1	1.2	46	1	2.2	2.4
White Nile	90	2	2.2	2.4	8	2	25.0	27.9
The Gezira	90	8	8.8	9.9	13	5	38.5	42.9
Blue Nile	270	16	5.9	6.6	57	14	24.5	27.4
River Nile	270	8	2.9	3.3	29	6	20.6	23.1
	810	35	Av. 4.2	Av. 4.8	153	28	Av. 22.3	24.9

### Result of risk factors

Statistical analysis of the potential risk factors for PTB showed a significant association between higher MAP infection rates and increased rainfall, as well as exposure to wild animals. No significant correlation was found between MAP infection and grazing system as well as exposure to other ruminants (Table 3).

**Table 3:** Estimation of risk factors associated with MAP infection in cattle in five states of the Sudan

Category	Groups	Odds ratio	Std. Err	Likelihood Ratio Chi-square	*P*-value	95% CI
Environmental condition	Long dry season High rainfall	1.5	24,048.5	5.0	0.02	0.0
Razing system	Semi-intensive Semi-extensive	0.8	2.3	1.24	0.3	0.3 1.2
Exposure to wild animals	Suspected Un suspected	1.5	24,048.5	5.0	0.02	0.0
Exposure to other ruminants	Continuously Sometimes	0.8	2.3	1.2	0.3	0.3 1.2

### Result of sequencing and phylogenetic analysis of MAP isolates

MAP isolates were detected in 5.6% (3/17) of the cattle faecal samples and confirmed through partial sequencing of the IS 1311. Analysis of the partial IS1311 sequences from the three MAP isolates revealed variations among them. Two isolates, Sona-C1 and Sona-C2, shared identical sequences, while Sona-C3 displayed a heterologous sequence. The Sona-C2 isolate demonstrated 99.12% identity to the MAP reference K-10 from the USA and showed high similarity to other worldwide strains. However, it exhibited lower identity (97.65%) to the MAP strain Case-3 from China (Appendix 2). In contrast, the Sona-C3 isolate displayed 99.57% identity to the MAP reference strain K-10 from the USA and aligned closely with other worldwide strains, but it showed lower (99.15%) identity to the MAP strain E1 from Egypt (Appendix 3). eThe phylogenetic tree constructed using the partial sequence MAP IS1311 of the Sudanese cattle isolates (Sona-C2 and Sona-C3) and the corresponding sequences of other strains worldwide is shown in Fig. 2. The Sudanese cattle isolates (Sona-C2 and Sona C3) clustered with MAP strains Telford, MAP 6756, MAP LN20, MAP S397, MAPK CN9/15 and MAP JIII-386 strains.

## Discussion

Paratuberculosis (PTB) is a contagious untreatable disease in ruminants characterized by granulomatous enteritis, caused by *Mycobacterium avium* subsp. *paratuberculosis* (MAP). The disease leads to great economic losses in livestock sector, particularly in dairy farms [6]. Although MAP infection in ruminants has been reported early in the Sudan [36, 37], Chaudhary et al., 1964; Fawi and Obied, 1964), it received little attention since then; studies on its prevalence remain scarce and mainly limited to Khartoum State [30, 31]. In a study conducted in the Sudan by our group [9], we reported the detection of MAP in 40% of human patients with various gastrointestinal diseases, including those who were diagnosed or suspected with inflammatory bowel disease (IBD). Despite that the study results did not link MAP positivity to a specific disease condition, it was noted that all MAP positive patients consumed milk, pointing out a potential public health risk. Therefore, this study aimed to better understand MAP distribution and PTB prevalence across the Sudan.

The serological assay (ELISA) and the molecular assay (RAA) were used for screening PTB in cattle. The average apparent and true seroprevalence of PTB were 5.0% and 8.5% at the animal level and 28.2% and 54.2% at the herd level, respectively. These results are comparable to those reported in Uganda by Ssekitoleko et al. [27], who found apparent seroprevalence of 3.2% and true seroprevalence of 4.9% at the animal level, with apparent and true herd-level prevalence of 43% and 42.8%, respectively. However, the apparent seroprevalence at the animal level in the present study was lower than that reported in other countries, in which ELISA was also used: 19.6% in Egypt [25], 19.7% in Saudi Arabia [23], 37.7% in West Bengal, India [18], and 14.2% in China [19]; the later authors also reported a higher herd-level seroprevalence of 66.7% in China. This disparity may be linked to differences in the production systems between these countries. Intensive production systems typically involve higher animal densities, which can increase the risk of MAP transmission through faecal contamination. In contrast, extensive systems generally reduce close contact among animals, thereby lowering the transmission rate [27, 38]. Additionally, variations in prevalence rates might be influenced by environmental factors [27, 39] and animal breed [13].

The average apparent and true molecular prevalence of PTB detected using RAA were 4.2% and 4.8% at the animal level, and 22.3% and 24.9% at the herd level, respectively. These values were lower than the PTB seroprevalence observed at both the animal and herd levels. Similarly, Ssekitoleko et al. [27], utilizing the same ELISA kit and RAA assays as in the current study, reported higher positivity rates for MAP antibodies compared to MAP DNA, with apparent and true molecular prevalence rates of 3.0% and 3.4% at the animal level, and 40.8% at the herd level, respectively. In contrast, Elsohaby et al. [23] and Navarro-Gonzalez et al. [40] reported a higher positivity rate for MAP DNA using PCR and qPCR, than for MAP antibodies using ELISA. It is worth mentioning that our results on PTB in small ruminants [38] found higher positivity rates for MAP DNA compared to MAP antibodies using the same assays as in this study. However, these discrepancies might be explained by the intermittent shedding of MAP in faeces [35]. Therefore, it is crucial to employ multiple screening tests with high sensitivity and specificity on more than one sample.

The findings of this study revealed variations in seroprevalence across different states, with higher rates observed in Blue Nile and the Gezira States compared to others. These differences might be influenced by factors such as environmental conditions, soil type, breed characteristics, interaction with wild animals and other ruminants and grazing practices. In Khartoum State, the apparent seroprevalence at the animal and herd levels was 3.3% and 11.4%, respectively. These results are slightly lower than those reported by Elmagzoub et al. [30] for the same state, documenting 6.3% prevalence at the animal level and 18.9% at the herd level. Our results are also lower than the findings of Mohammed et al. [31], who reported 10.2% and 66.7% prevalence for animal and herd levels, respectively. These comparisons may suggest a decrease of seroprevalence of PTB over time in Khartoum State. Possible reasons could include advancements and expansion of veterinary services and overall improvement in herd health management practices. This is specific for this State, where the economic importance of dairy cattle is high.

The spread of MAP infection is influenced by various factors, many of which have been identified as potential risk factors [13–15, 41–43]. All animals in the surveyed states of this study were subject to the local environmental conditions and associated stresses. High rainfall was found to significantly (*p* < 0.05) affect disease prevalence at herd level. This is likely because rain and flood water spread faecal material of infected animals from contaminated areas across pastures and into water sources, ultimately carrying infection to remote areas [44, 45]. Furthermore, prolonged moisture in the soil may enhance the survival of MAP, thereby increasing the risk of environmental transmission during and after the rainy season. However, apparently contrasting findings were reported by Ssekitoleko et al. [27] that MAP infection was higher in areas which experienced prolonged dry spells compared to short dry periods in Uganda. Under dry conditions, desiccated faeces become airborne and can be carried with dust particles, facilitating the windborne spread of MAP. Additionally, the dry season often leads to increased animal congregation around limited water and feed sources, which may promote closer contact and enhance faecal-oral transmission, especially under stressful conditions. These findings suggest that MAP transmission can be facilitated under both rainy and dry conditions, depending on specific environmental condition. Nevertheless, the high temperatures and extended dry seasons, typical of many areas in the Sudan, may suppress MAP survival. Many studies indicated that desiccation, ultraviolet radiation and high soil temperatures can negatively impact MAP persistence in the environment as reviewed by Elliott et al. [46]. These environmental stressors likely contribute to the reduced prevalence of PTB observed in some regions during dry months as shown in Khartoum State (2.4%). In addition, soil characteristics also play a critical role in modulating MAP survival and dissemination. Some studies reported that MAP may be inactivated by soil drying, acidification, ammonia exposure, and low iron content factors that vary significantly across Sudan’s agro-ecological areas and other countries [47, 48]. However, Dhand et al. [49] noted that MAP prevalence was higher in cattle and sheep raised on acidic soils, suggesting that certain soil types might actually favor MAP persistence, especially if moisture and organic matter are retained in the upper soil layers.

Overall, the variation of PTB prevalence across different States in the Sudan may reflect the complex interplay of climatic and environmental factors influencing pathogen survival and transmission dynamics, especially with the regional variability in rainfall intensity, soil composition and temperature extremes.

The grazing system in the central region was semi-intensive (Khartoum, Gezira and some parts of White Nile), and semi-extensive in the Blue Nile and parts of White Nile and the River Nile States. In semi-intensive farming systems, it is common practice to separate calves from mature cattle, whereas segregation of healthy and sick animals is generally not implemented. Cattle on open pastures frequently encounter herds from other ruminant species or come into contact with their faeces. However, this study did not find a significant link between grazing systems or interaction with other ruminants and MAP infection. Moreover, previous studies have indicated that open pasture or extensive systems, which promote greater animal interaction, may be more effective in limiting the spread of MAP infection compared to intensive systems (Liu et al., 2017). In the Blue Nile State, where part of the largest national wildlife reserve park, El Dinder Park, is located contact with wild animals is expected. This could be supported by Mohammed et al. [50] who observed illegal presence of large cattle herds in El Dinder. Notably, this study revealed a significant association (p < 0.05) between contact with wild animals and MAP infection. A parallel investigation for paratuberculosis conducted by the same authors in wildlife in El Dinder Park showed a high prevalence rate (33.3%) among wild animals (unpublished data). This aligns with numerous studies [51–55], which have identified co-grazing and contact with the faeces of infected wild animals as a potential risk factor for introducing MAP into herds. This interaction may serve as a transmission bridge, as wild ruminants can act as MAP reservoirs or mechanical carriers, particularly in extensive grazing systems where natural habitats overlap with livestock areas. These results highlight the importance of wildlife interaction control into PTB prevention strategies.

In the phylogeny tree, Sudanese cattle MAP isolates Sona_C2 and Sona_C3 clustered together with MAP S397 strain from the USA, identified as Type S (sheep-type) [56] forming a subclade in the clade formed by Indian, Egyptian, some East Asian and some Europian countries strains such as Telford from Australia, MAP 386 from Germany, and MAP 6756 from New Zealand, all of which were isolated from sheep and classified as type S strains [56–59]. Based on these findings, the Sudanese cattle isolates (Sona-C2 and C3) are suggested to be classified as type S (I/III) strains. This result can be supported by possibility of interspecies transmission [60]. It is worth noting that the livestock production systems in the Sudan often involve raising multiple animal species together on the same farm or pasture. This practice frequently combines small ruminants with cattle [29]. Furthermore, PTB is endemic and highly prevalent in many developed countries [61]. It is likely that PTB was initially introduced to Sudan through the importation of European cattle breeds, primarily Friesian, to upgrade the local breeds (Babiker Abbas, personal communication). This could explain the close genetic similarity observed between MAP isolates from the Sudan and certain strains from European countries. Friesian cattle are also common in New Zealand (Elischer, 2014), where type S strains are more prevalent in cattle than type C strains (Verdugo et al., 2014). This could also explain the close relatedness of the Sudanese isolates to MAP strain 6756 from New Zealand. In contrast, the Sudanese MAP isolates were distantly related to those from Egypt (E1 and E93 strains), despite the geographical proximity. This distant relatedness could be due to absence of interaction between cattle in the Sudan and those in Egypt. In addition, Holstein dairy cows in Egypt were imported from the USA, so the MAP strains from Egypt are closely related to the reference strain K10 from the USA. Notably, the well-characterized MAP reference strain K-10 is a type C (II) strain [62], originally isolated from the USA, is located in the phylogeny tree in a separate clade formed by other type C strains. Therefore, the Sudanese isolates share a distant common ancestor with K-10.

Not only genotypic features provide a strong evidence that Sudanese cattle MAP isolates are sheep-type (Type S), but also phenotypic characteristics of Sudanese cattle MAP isolates provide another evidence. They were slow growing and formed pin-white colonies, the typical features of sheep-type strains. Moreover, our data on other MAP isolates from sheep, goats, camels, as well as from humans, suggests that the sheep-type (Type S) is common in the Sudan [63]. However, whole genome analysis would provide more insights into the lineages of sheep-type MAP circulating in the Sudan to enhance our understanding of MAP transmission dynamics.

## Conclusions

This study provides insights into the prevalence and risk factors associated with paratuberculosis (PTB) in cattle across different regions of the Sudan. The findings revealed a moderate prevalence of PTB, with a significant association between *Mycobacterium avium* subspecies *paratuberculosis* (MAP) infection and the risk factors of rainfall and the presence of wild animals. The study also highlights the importance of using multiple diagnostic methods, such as serological and molecular tests, to accurately assess MAP prevalence, given the challenges of detecting the infection through intermittent faecal shedding and varying antibody responses.

The close genetic relationship between Sudanese MAP isolates and strains from Europe and New Zealand points to the possible introduction of PTB to the Sudan through imported European cattle, particularly Friesian.

Given the significant economic and public health risks posed by PTB, especially in dairy cattle, this study highlights the need for increased awareness of the disease, enhanced surveillance, and the implementation of effective control measures to prevent its spread. Further research is essential to better understand the dynamics of PTB transmission and to explore the influence of environmental and genetic factors on MAP distribution in the Sudan.

## Appendix 1

Table 1

**Table 1:** Check list for some possible risk factors associated with presence of cattle paratuberculosis in Sudan

Category	Groups
Environmental condition	High rainfall Long dry period
Grazing system	Semi-intensive Semi-extensive
exposure to wild animals	Suspected Unsuspected
exposure to other ruminants	continuously sometimes

## Appendix 2

Table 2

**Table 2:** Sudanese cattle MAP isolate (Sona-C2), related to other strains elsewhere

Strain	Identity %	Country	GenBank Accession number	Animal Host
MAPK_JB16/15	99.12%	South Korea	CP033911.1	Bovine
DSM 44135	99.12%	Germany	CP053068.1	Bovine
Telford	99.12%	Australia	CP033688.1	Sheep
LN20	99.12%	Canada	CP113485.1	Pig
K-10	99.12%	USA	CP106873.1	Bovine
Isolate Case-3	97.65%	China	MW546851.1	Goat

## Appendix 3

Table 3

**Table 3:** Sudanese cattle MAP isolate (Sona-C3), related to other strains elsewhere

_Strain_	Identity %	Country	GenBank Accession Number	Animal Host
MAPK_JB16/15	99.57%	South Korea	CP033911.1	Bovine
Telford	99.57%	Australia	CP033688.1	Sheep
K-10	99.57%	USA	CP106873.1	Bovine
PICSAR77 isolate	99.57%	France	CP091844.1	Bovine
MAP E1	99.15%	Egypt	CP010113.1	

## Appendix 4

### Alignment of the partial sequence of the IS1311 gene of *Mycobacterium avium *subsp. *paratuberculosis* isolated from Sudanese cattle (Sona-C2)

*Mycobacterium avium subsp. paratuberculosis* strain Telford chromosome, complete genome.

## Appendix 5

### Alignment of the partial sequence of the IS1311 gene of *Mycobacterium avium *subsp. *paratuberculosis* isolated from Sudanese cattle (Sona-C3)

*Mycobacterium avium subsp. paratuberculosis* strain Telford chromosome, complete genome.
